# Short-term effect of dopaminergic medication on speech in early-stage Parkinson’s disease

**DOI:** 10.1038/s41531-022-00286-y

**Published:** 2022-03-07

**Authors:** Tereza Tykalova, Michal Novotny, Evzen Ruzicka, Petr Dusek, Jan Rusz

**Affiliations:** 1 Department of Circuit Theory, Faculty of Electrical Engineering, Czech Technical University in Prague, Prague, Czech Republic; 2 Department of Neurology and Centre of Clinical Neuroscience, First Faculty of Medicine, Charles University, Prague, Czech Republic

**Keywords:** Parkinson's disease, Parkinson's disease

## Abstract

The effect of dopaminergic medication on speech has rarely been examined in early-stage Parkinson’s disease (PD) and the respective literature is inconclusive and limited by inappropriate design with lack of PD control group. The study aims to examine the short-term effect of dopaminergic medication on speech in PD using patients with good motor responsiveness to levodopa challenge compared to a control group of PD patients with poor motor responsiveness. A total of 60 early-stage PD patients were investigated before (OFF) and after (ON) acute levodopa challenge and compared to 30 age-matched healthy controls. PD patients were categorised into two clinical subgroups (PD responders vs. PD nonresponders) according to the comparison of their motor performance based on movement disorder society-unified Parkinson’s disease rating scale, part III. Seven distinctive parameters of hypokinetic dysarthria were examined using quantitative acoustic analysis. We observed increased monopitch (*p* > 0.01), aggravated monoloudness (*p* > 0.05) and longer duration of stop consonants (*p* > 0.05) in PD compared to healthy controls, confirming the presence of hypokinetic dysarthria in early PD. No speech alterations from OFF to ON state were revealed in any of the two PD groups and speech dimensions investigated including monopitch, monoloudness, imprecise consonants, harsh voice, slow sequential motion rates, articulation rate, or inappropriate silences, although a subgroup of PD responders manifested obvious improvement in motor function after levodopa intake (*p* > 0.001). Since the short-term usage of levodopa does not easily affect voice and speech performance in PD, speech assessment may provide a medication state-independent motor biomarker of PD.

## Introduction

Hypokinetic dysarthria is a common symptom of Parkinson’s disease (PD), manifesting in up to 90% of patients during the disease course^1^. It is a complex motor speech disorder characterised mainly by monopitch, monoloudness, reduced stress, imprecise articulation, inappropriate silences, variable rate and harsh voice quality^2,3^. Hypokinetic dysarthria can occur since the prodromal stage of PD^4^ and becomes more severe as the disease progresses, potentially resulting in loss of functional communication^1^.

Although dopaminergic treatment provides beneficial effects on motor manifestations, mixed results have been reported regarding the effect of levodopa challenge on speech in PD^5–20^. Yet, the available evidence about the effect of levodopa on speech in PD is limited due to small sample sizes, heterogeneity of studied population, lack of concise and valid speech recording and analysis methodology, and lack of motor scores. After levodopa intake, improvements in voice quality^13,15,17^, loudness^11,19^, speaking rate^14^, speech dysfluency^12^, respiration^5,8^ and overall speech intelligibility^8^ have been reported, while other studies found no significant changes in voice quality^5,6,9^, loudness^5,10,18^, speaking rate^7,9,18^, vowel articulation^11,18^, consonant articulation^20^, monopitch^5,9,18^ or respiration^9,16^. This inconsistency may be caused, at least partially, by different disease duration and motor severity of investigated PD patients. While most of the studies focused on advanced^5–10,12,19^ or mixed^11,13,15–17,20^ stages, little is known about the acute effect of levodopa on speech in early PD patients^14,18^. Nevertheless, the stage of the disease may play an important role in a short-term levodopa response^21,22^. As PD progresses, the magnitude of short-duration levodopa response increases and fluctuation appears^21,22^ which both might significantly influence speech. Therefore, evidence about the acute effect of levodopa on speech in early PD patients is pivotal to provide more insights into the pathophysiology of dysarthria in PD. Another limitation of previous research likely contributing to inconclusive results^5–20^ is the lack of stratification of PD patients according to their cardinal motor symptoms’ response to acute levodopa challenge, leading to the inclusion of patients with weak or no motor response to levodopa challenge. However, one might hardly expect that PD patients with minimal change in motor function will manifest significant improvement in their speech performance.

The aim of the current study was to examine the short-term effect of dopaminergic medication on speech in early PD using patients with good motor responsiveness compared to a control group of PD patients with poor motor responsiveness to acute dopaminergic challenge.

## Results

**Fig. 1:**
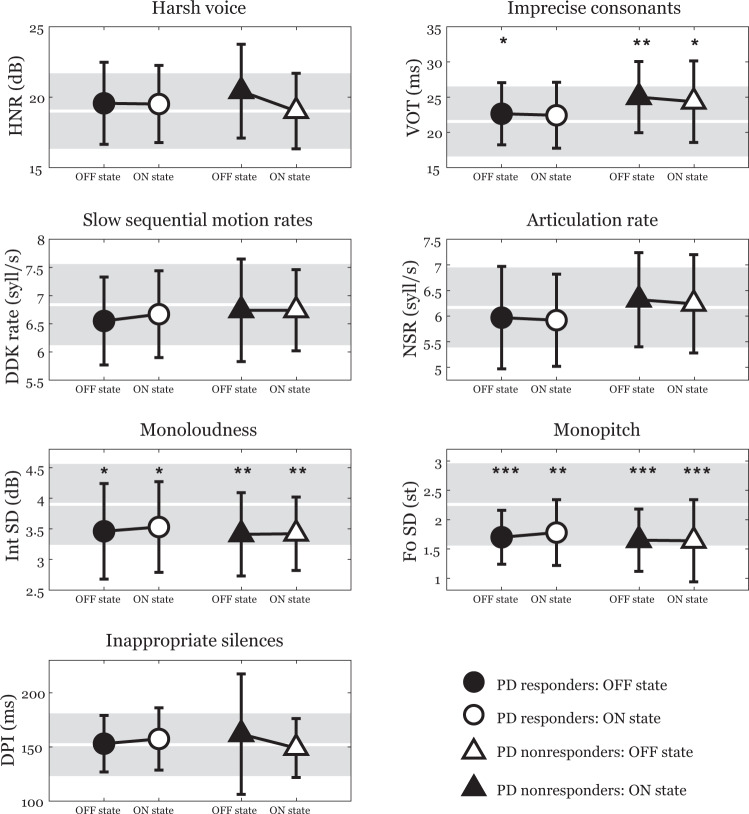
Results of acoustic analyses across PD responders and PD nonresponders subgroups in both OFF and ON condition. Symbols represent mean values and error bars represent SD values. The performances of healthy controls are depicted by a shaded area. Comparing subgroups of PD responders and PD nonresponders, no significant differences for effect of GROUP, MEDICATION or GROUP × MEDICATION were found. Statistically significant differences between PD subgroups and healthy controls: *p < 0.05; **p < 0.01; ***p < 0.001. PD Parkinson’s disease, HNR harmonics-to-noise ratio, VOT voice onset time, DDK rate diadochokinetic rate, NSR net speech rate, Int SD standard deviation of speech intensity, F0 SD standard deviation of the fundamental frequency, DPI duration of pause intervals.

Figure 1 shows the results of acoustic analyses across PD responders and PD nonresponders in both OFF and ON conditions.

Compared to healthy controls, we revealed increased VOT in all PD subgroups (*p* > 0.05) except for PD responders in ON state (*p* = 0.49), decreased Int SD in all PD subgroups (*p* > 0.05), and decreased F0 SD in all PD subgroups (*p* > 0.01). For the remaining four acoustic parameters, no statistically significant differences between PD and healthy controls were found.

Comparing subgroups of PD responders and PD nonresponders with respect to affected speech dimensions, for imprecise consonants articulation measured via VOT, no main effect of GROUP [*F*(1, 58) = 3.2, *p* = 0.08, *η2* = 0.05], MEDICATION [*F*(1, 58) = 0.8, *p* = 0.37, *η2* = 0.01] or GROUP × MEDICATION [*F*(1, 58) = 0.2, *p* = 0.67, *η2* = 0.003] interaction was detected. For monoloudness measured via Int SD, no main effect of GROUP [*F*(1, 58) = 0.2, *p* = 0.64, *η2* = 0.004], MEDICATION [*F*(1, 58) = 0.4, *p* = 0.55, *η2* = 0.006] or GROUP × MEDICATION [*F*(1, 58) = 0.2, *p* = 0.67, *η2* = 0.003] interaction was observed. Finally, for monopitch measured via F0 SD, there was found no main effect of GROUP [*F*(1, 58) = 0.6, *p* = 0.45, *η2* = 0.01], MEDICATION [*F*(1, 58) = 1.3, *p* = 0.26, *η2* = 0.02] or GROUP × MEDICATION [*F*(1, 58) = 1.8, *p* = 0.19, *η2* = 0.03] interaction. In addition, we did not detect any significant differences for effect of GROUP, MEDICATION or GROUP × MEDICATION for the remaining four acoustic parameters investigated.

## Discussion

This study determines the short-term effect of dopaminergic medication on speech in PD and its association with motor responsiveness to the levodopa challenge. To clarify the previous inconclusive findings^5–20^, a subgroup of PD patients with a good motor response as well as a control group of PD patients with none or weak change on MDS-UPDRS III after acute levodopa challenge were investigated in a practically defined OFF medication state and then retested 60–90 min later in their best ON medication state. No effect of levodopa was found on voice and speech performances in any PD subgroup, despite the PD responders subgroup manifested obvious improvement in motor function after levodopa intake. The strength of this study is that speech features were evaluated in homogeneous cohort of early PD patients using objective, fully-automated acoustic analysis that is sensitive to subtle speech changes^4^.

In particular, we observed increased monopitch, aggravated monoloudness and longer duration of stop consonants in early PD compared to healthy controls, confirming the presence and distinctive patterns of hypokinetic dysarthria in early PD^4,18,23^. However, no speech changes after acute levodopa intake were revealed in any of these speech dimensions or other dimensions investigated including harsh voice, slow sequential motion rates, articulation rate or inappropriate silences. These findings are in accordance with previous research investigating early PD^18^ as well as with the majority of prior studies focused on advanced PD^6,7,9,10^, suggesting that speech could be considered as a PD symptom resistant to short-term levodopa treatment.

Interestingly, probably the most effort has been put into the investigation of the short-term effect of levodopa on voice quality; however, the results remain inconclusive. Whereas some authors found jitter, shimmer, HNR, cepstral peak prominence or pitch breaks to be unresponsive to short-term dopaminergic stimulation^5,6,9^, others were pointing to a dopamine-related amelioration in phonatory parameters^13,15,17^. Cushnie-Sparrow et al.^6^ divided patients into two groups based on voice quality severity perceptually evaluated by three listeners and found that only PD patients with poor voice quality off-medication showed improvements in voice quality on-medication, while those with better voice quality did not. Our cohort of early-stage PD patients with relatively mild impairment of voice quality did not show any change of voice quality (i.e. HNR) between ON and OFF state. Nevertheless, it should be mentioned that amelioration of voice quality after acute levodopa challenge was also not found in late-stage PD patients^5,9^, who commonly suffer from dysphonia.

One potential explanation for the poor short-term effect of dopaminergic therapy on speech in early PD is potential masking by the long-duration response of levodopa treatment, which is being more prominent in the early stages and accounts for the stable response seen in the ‘honeymoon’ treatment period^21^. It has been shown that a prolonged washout of 15 days from chronically administered levodopa may be needed for evaluation of actual short-term response^22^. This assumption is in agreement with a recent longitudinal study examining a large sample of de novo untreated PD subjects 1 year after levodopa admission, which reported that most patients improved or maintained their initial speech performance^24^. Based on these findings, one might hypothesise that stable long-term dopaminergic stimulation has an apparent positive effect on speech^23,24^, whereas 1-day dopamine medication withdrawal may simply be a too short period to achieve an effective OFF state with regards to speech pathology.

However, the different pathophysiologic mechanisms underlying individual speech components may also play a role in dopaminergic responsiveness. The beneficial long-term effect of levodopa was notable mainly in the subtype of patients with predominant dysphonia^24^, supposedly having the most severe laryngeal rigidity caused by dopaminergic involvement. Contrary, a subtype with prevailing articulation deficits, which also had the most severe cognitive and axial gait dysfunction, did not respond to long-term dopaminergic medication^24^. This finding indirectly suggests that hypokinetic articulation might be attributed to the degeneration of non-dopaminergic pathways. In accordance with this assumption, a former study reported that aggravation of dysarthria during PD progression results mainly from the increasing severity of cerebral non-dopaminergic lesions^25^. Further evidence comes from PD patients with bilateral subthalamic nucleus deep brain stimulation, where the severity of the residual parkinsonian speech score in ON medication state was predictive of a poor postoperative outcome, likely due to the presence of non-dopaminergic lesions within the basal ganglia, which would not respond to medication or, thus, to stimulation^26^.

It is also likely that the differing motor responsiveness to acute dopaminergic challenge depends on whether patients reached a hypodopaminergic state after short-term medication withdrawal. Indeed, the PD responders subgroup had a higher MDS-UPDRS III score in the OFF state and a higher MDS-UPDRS IV score (although there was only a trend for statistical significance in the latter) compared to the PD nonresponders subgroup suggesting that the PD responders subgroup has incipient motor fluctuations. The stability of speech quality during the hypodopaminergic state in the PD responders subgroup might be explained by differing dopaminergic sensitivity of brain systems responsible for speech and limb function^27,28^.

Findings of our study highlight new opportunities regarding the usage of speech assessment as a potential PD biomarker. Neuromodulatory or even neuroprotective therapies for PD are currently under development, raising the question of how we should define and measure disease progression^29^. Ideal progressive biomarker must be adaptable to the different stages of the disease, broadly available, reasonably priced and easy to use. Compared to bradykinesia or rigidity measures which reflect functional presynaptic dopaminergic activity^29,30^, our results indicate that evaluation of speech impairment is more stable and not easily influenced by short-term variations in dopaminergic medication dose. Therefore, speech could be used as a complementary motor marker of disease severity that may be less affected by fluctuating plasmatic levels of dopaminergic drugs. In addition, speech impairment has been observed from prodromal stages of PD^4^ with progression documented via longitudinal studies from de novo^24^, through early^4^, to late PD stages^31,32^. Given this aggregate evidence and the fact that vocal assessment is rapid, cheap, easy to administer, scalable to large populations, and can be performed remotely, probably even by smartphones^33^, it has the potential to provide an ideal progressive biomarker of PD^29^.

One potential limitation of this study is that patients were given their usual dose of levodopa medication after overnight withdrawal, while it is possible that this washout period may lead to an under-medication in a defined ON medication state. Nevertheless, neither the studies using a standard dose of levodopa equal to 200^18^ or 300 mg^6^ nor the studies using 30^5^ or 50% higher daily dose^9^ of usual levodopa morning intake revealed significant differences in speech production between ON and OFF state.

In conclusion, our results emphasise that short-term usage of dopaminergic medication and mild motor fluctuations have a little effect on voice and speech performance in early-stage PD, despite obvious improvement in motor function after levodopa intake. Future studies should further explore the potential effect of dopaminergic medication on speech using detailed monitoring of levodopa pharmacokinetics^34^.

## Methods

### Participants

From 2017 to 2020, a total of 60 consecutive patients who fulfilled the clinical diagnosis of PD based on the Movement Disorder Society clinical diagnostic criteria for PD^35^ were enroled. The inclusion criteria for PD were as follows: (i) usage of a stable dopaminergic medication dose in last 4 weeks, mainly consisting of levodopa and/or dopamine agonist, (ii) Hoehn & Yahr stage 1–2 in the defined OFF medication state and (iii) no history of communication or significant neurological disorders unrelated to PD. All patients were investigated in the morning in a practically defined OFF medication state (obtained after 12 h levodopa and/or 48 h dopamine agonist withdrawal) and then retested in the defined ON medication state (obtained 60–90 min after the administration of the patient’s usual levodopa and/or dopamine agonists morning intake). Neurological examination was performed using the Movement Disorder Society-Unified Parkinson’s Disease Rating Scale (MDS-UPDRS)^36^ and Montreal Cognitive Assessment (MoCA)^37^. The daily doses of dopaminergic medication were recalculated to l-dopa equivalent^38^.

PD patients were categorised into two clinical subtypes, PD patients with good motor responsiveness to short-term dopaminergic therapy (hereafter, PD responders) and PD patients with none or weak motor responsiveness to short-term dopaminergic therapy (hereafter, PD nonresponders), according to the comparison of their performance in MDS-UPDRS III total in OFF vs. ON condition. Based on the previous literature^39,40^, the cut-off values for differentiation between subgroups were defined as follows:

(i) PD responders: the minimum percentage change of MDS-UPDRS III from OFF to ON condition ≥20% and minimal clinically important improvement on MDS-UPDRS III from OFF to ON condition ≥4;
(ii) PD nonresponders: the maximum percentage change of MDS-UPDRS III from OFF to ON condition <20% and maximal clinically important improvement on MDS-UPDRS III from OFF to ON condition ≤3.

The PD responders subgroup consisted of 30 subjects (21 men, 9 women), and the PD nonresponders subgroup included 30 subjects (19 men, 11 women). No statistically significant differences were found between the PD responders and PD nonresponders subgroups for age, disease duration, medication dose, cognitive status, speech severity or motor severity in ON medication state (*p* = 0.24–0.82). The PD responders subgroup showed a higher MDS-UPDRS III score in the OFF medication state (*p* = 0.01) and a greater decrease of motor severity from OFF to ON condition (*p* < 0.001) than PD nonresponders. Disease duration was estimated based on the self-reported occurrence of the first motor symptoms. Perceptual speech severity was assessed using the speech item 3.1 score of the MDS-UPDRS III. Detailed patient characteristics are summarised in Table 1.

**Table 1:** Clinical characteristics of PD patients.

	PD responders	PD nonresponders	*p* value (independent *t*-test)
	(*n* = 30; 21 men, 9 women)	(*n* = 30; 19 men, 11 women)	
	Mean/SD (range)	Mean/SD (range)	
Age (years)	61.0/12.3 (35−79)	63.3/10.9 (43−82)	0.45
PD symptom duration (years)	3.8/2.0 (1.3−8.1)	3.3/1.6 (1.5−6.9)	0.64
Hoehn & Yahr scale	2.0/0.0 (2−2)	2.0/0.2 (1−2)	0.35
MDS-UPDRS III OFF	29.0/10.4 (15−56)	22.6/9.0 (12−45)	**0.01**
MDS-UPDRS III ON	18.9/8.6 (6−42)	21.3/8.8 (11−45)	0.28
MDS-UPDRS III speech item OFF	0.6/0.5 (0−1)	0.6/0.6 (0−2)	0.82
MDS-UPDRS III speech item ON	0.5/0.5 (0−1)	0.6/0.6 (0−2)	0.48
Δ (MDS-UPDRS III OFF - MDS-UPDRS III ON)	10.2/5.3 (5−23)	1.2/1.4 (−2−3)	**<0.001**
Change of MDS-UPDRS III from OFF to ON (%)	35.5/14.2 (21−79)	7.4/4.4 (0−19)	**<0.001**
MDS-UPDRS IV	0.8/1.9 (0−6)	0.1/0.5 (0−2)	0.09
L-dopa equivalent (mg/day)	527/290 (240−1740)	443/264 (50−1125)	0.24
MoCA	25.1/2.4 (20−29)	25.8/2.9 (18−30)	0.37

Bold values indicates statistical significant *P* values.

*PD* Parkinson’s disease, *MDS-UPDRS* movement disorder society-unified Parkinson disease rating scale, *MoCA* Montreal cognitive assessment.

The healthy control group consisted of 30 subjects (20 men, 10 women) of comparable age (mean 62.3, standard deviation 10.3, range 35–81 years) with no history of neurological or communication disorders. All participants were Czech native speakers.

Each participant provided written informed consent to the neurological examination and recording procedure. The study received approval from the Ethics Committee of the General University Hospital in Prague, Czech Republic and has therefore been performed in accordance with the ethical standards established in the 1964 Declaration of Helsinki.

### Speech examination

Speech recordings were performed in a quiet room with a low ambient noise level using a head-mounted condenser microphone (Beyerdynamic Opus 55, Heilbronn, Germany) placed ~5 cm from the corner of the subject’s mouth. Speech signals were sampled at 48 kHz with 16-bit resolution. All participants were asked to perform three speaking tasks: (i) sustained phonation of the vowel /a/ per one breath for as long and steadily as possible, (ii) fast repetition of syllables /pa/-/ta/-/ka/ at least ten times per one breath and (iii) a standardised reading passage composed of 80 words. These three speaking tasks were selected because they can provide most of the information necessary for the accurate description and interpretation of motor speech disorders in PD^3,41^. All speaking tasks were performed twice for each participant.

### Acoustic analysis

Based on the results of a previous multi-centric study investigating a large sample of early-stage PD patients^4^, we selected seven distinct speech parameters that allow quantitative objective acoustic assessment and correspond to the perceptual description of the main patterns of hypokinetic dysarthria^2,3^. In particular, *harsh voice* was examined using the harmonics-to-noise ratio (HNR) via sustained phonation. *Imprecise consonants* were assessed using the voice onset time (VOT) via fast syllable repetition. *Slow sequential motion rates* were evaluated using the diadochokinetic rate (DDK rate) via fast syllable repetition. *Articulation rate* was calculated through the net speech rate (NSR) via reading passage. *Monoloudness* was assessed using the standard deviation of intensity contour (Int SD) via reading passage. *Monopitch* was evaluated using the standard deviation of pitch contour (F0 SD) via reading passage. *Prolonged pauses* were computed using the duration of pause intervals (DPI) via reading passage. We averaged the final values used for the statistical analyses across two repetitions of speaking tasks to provide greater speech assessment stability. The definitions and pathophysiological interpretation of acoustic parameters are summarised in Table 2. Comprehensive algorithmic details on individual acoustics features have been reported previously^42^. In addition, the accuracy of algorithms for the identification of temporal intervals, pitch sequences and glottal cycles has been thoroughly tested in previous studies^42–44^. All analyses were performed in MATLAB (MathWorks, Natick, MA).

**Table 2:** Overview of applied acoustic measures.

Deviant speech dimension	Acoustic	Definition	Pathophysiological interpretation
(speaking task)	feature		with respect to hypokinetic dysarthria
Harsh voice	HNR	Harmonics-to-noise ratio, defined as the amount of noise in the speech signal.	Reduced rate of airflow and improper control of vocal folds causes increased turbulent noise.
(sustained phonation)			
Imprecise consonants	VOT	Voice onset time, defined as the length of the entire consonant from initial burst to vowel onset.	Hypokinesia causes slowing of lip and tongue movements, leading to a longer time required to pronounce individual consonants.
(syllable repetition)			
Slow sequential motion rates	DDK rate	Diadochokinetic rate, defined as the number of syllable vocalisations per second.	Hypokinesia of speech apparatus makes the movements of articulators slower.
(syllable repetition)			
Articulation rate	NSR	Net speech rate, defined as the total number of syllables divided by the total duration of speech after removal of pauses.	Impaired control of orofacial muscles leads to a decrease in speech rate.
(reading passage)			
Monoloudness	Int SD	Standard deviation of speech intensity contour extracted from voiced segments.	Hypokinesia leads to the decreased amplitude of respiratory and thyroarytenoid muscles.
(reading passage)			
Monopitch	F0 SD	Standard deviation of fundamental frequency contour converted to semitone scale.	Hypokinesia causes the reduced amplitude of vocal cord movements, leading to glottal incompetence.
(reading passage)			
Inappropriate silences	DPI	Duration of pause intervals, defined as the median length of pause intervals.	Hypokinesia of speech apparatus makes initiating speech difficult, leading to prolonged pause intervals.
(reading passage)			

### Statistical analysis

The Kolmogorov–Smirnov test for independent samples revealed normal distribution in all parameters. To determine the effect of short-term dopaminergic medication on speech, we applied a 2 × 2 repeated measure analysis of variance (RM-ANOVA) with GROUP (PD responders, PD nonresponders) being treated as between-group factors and MEDICATION (OFF state, ON state) being treated as a within-group factor. Post hoc significance was assessed by the Fisher’s least-squares difference. The two-sample *t*-test was used to assess group differences between healthy controls and PD subgroups. All statistical analyses were performed using MATLAB (MathWorks, Natick, MA, USA).

### Reporting Summary

Further information on research design is available in the Nature Research Reporting Summary linked to this article.

## Supplementary information

Reporting Summary Checklist

## Data Availability

Individual participant data that underlie the findings of this study are available upon reasonable request from the corresponding author. The speech data are not publicly available due to their content of information that could compromise the privacy of study participants.

## Code availability

The analyses were performed using a publicly available Dysarthria Analyzer (Czech Technical University in Prague, available at http://dysan.cz/).
