# Supplementary material for: Short-term effect of dopaminergic medication on speech in early-stage Parkinson’s disease
Source: NPJ Parkinsons Dis. 2022 Mar 7;8:22. doi: 10.1038/s41531-022-00286-y (PMC8901688; doi:10.1038/s41531-022-00286-y)
Supplement: Supplementary file 1 — Reporting Summary Checklist [file 41531_2022_286_MOESM1_ESM.pdf]

nature portfolio

Corresponding author(s): Tereza Tykalova
Last updated by author(s): Jan 25, 2022

Reporting Summary

Nature Portfolio wishes to improve the reproducibility of the work that we publish. This form provides structure for consistency and transparency in reporting. For further information on Nature Portfolio policies, see our Editorial Policies and the Editorial Policy Checklist.

Statistics

For all statistical analyses, confirm that the following items are present in the figure legend, table legend, main text, or Methods section.

n/a	Confirmed	
☐	☒	The exact sample size (n) for each experimental group/condition, given as a discrete number and unit of measurement
☐	☒	A statement on whether measurements were taken from distinct samples or whether the same sample was measured repeatedly
☐	☒	The statistical test(s) used AND whether they are one- or two-sided<br>*Only common tests should be described solely by name; describe more complex techniques in the Methods section.*
☐	☒	A description of all covariates tested
☐	☒	A description of any assumptions or corrections, such as tests of normality and adjustment for multiple comparisons
☐	☒	A full description of the statistical parameters including central tendency (e.g. means) or other basic estimates (e.g. regression coefficient) AND variation (e.g. standard deviation) or associated estimates of uncertainty (e.g. confidence intervals)
☐	☒	For null hypothesis testing, the test statistic (e.g. F, t, r) with confidence intervals, effect sizes, degrees of freedom and P value noted<br>*Give P values as exact values whenever suitable.*
☒	☐	For Bayesian analysis, information on the choice of priors and Markov chain Monte Carlo settings
☒	☐	For hierarchical and complex designs, identification of the appropriate level for tests and full reporting of outcomes
☐	☒	Estimates of effect sizes (e.g. Cohen's d, Pearson's r), indicating how they were calculated

Our web collection on statistics for biologists contains articles on many of the points above.

Software and code

Policy information about availability of computer code

Data collection	No software was used.
Data analysis	All analyses were performed in MATLAB® (MathWorks, Natick, MA).

For manuscripts utilizing custom algorithms or software that are central to the research but not yet described in published literature, software must be made available to editors and reviewers. We strongly encourage code deposition in a community repository (e.g. GitHub). See the Nature Portfolio guidelines for submitting code & software for further information.

Data

Policy information about availability of data

All manuscripts must include a data availability statement. This statement should provide the following information, where applicable:

- Accession codes, unique identifiers, or web links for publicly available datasets
- A description of any restrictions on data availability
- For clinical datasets or third party data, please ensure that the statement adheres to our policy

Individual participant data that underlie the findings of this study are available upon reasonable request from the corresponding author. The speech data are not publicly available due to their contain of information that could compromise the privacy of study participants.

Field-specific reporting

Please select the one below that is the best fit for your research. If you are not sure, read the appropriate sections before making your selection.

☐ Life sciences ☒ Behavioural & social sciences ☐ Ecological, evolutionary & environmental sciences

For a reference copy of the document with all sections, see nature.com/documents/nr-reporting-summary-flat.pdf

Behavioural & social sciences study design

All studies must disclose on these points even when the disclosure is negative.

Study description

Quantitative cross-sectional study.

Research sample

This study is based on a large sample of 60 patients (40 men, 20 women) with mean age of 62.2 (standard deviation 11.6, range 35-82) years, considering that only early-stage Parkinson's disease (Hoehn & Yahr stage 1-2 in the defined OFF medication state) was considered. The healthy control group consisted of 30 subjects (20 men, 10 women) of comparable age (mean 62.3, standard deviation 10.3, range 35–81 years) with no history of neurological or communication disorders.

Sampling strategy

An ad-hoc power analysis based on two-way repeated analysis of variance with two covariates (GROUP and MEDICATION) indicated a recommended minimum overall sample size of 36 for 4 groups (i.e., a minimum sample size of 8 per one group), given expected medium effect size (Cohen's f of 0.25) with the error probability α set at 0.05 and a false negative rate β set at 0.2 (i.e., power of 0.8). We included sample size of 60 subjects for 4 groups.

Data collection

Speech recordings were performed in a quiet room with a low ambient noise level using a head-mounted condenser microphone (Beyerdynamic Opus 55, Heilbronn, Germany) placed approximately 5 cm from the corner of the subject's mouth. Speech signals were sampled at 48 kHz with 16-bit resolution. Researcher was blind to the study hypothesis during data collection.

Timing

From 2016 to 2021.

Data exclusions

No data were excluded from the analyses.

Non-participation

No participants fullfilling inclusion/exlusion criteria stated within manuscript dropped from the analyses.

Randomization

Parkinson's disease patients were categorised into two clinical subtypes, Parkinson's disease patients with good motor responsiveness to short-term dopaminergic therapy (hereafter, PD responders) and Parkinson's disease patients with none or weak motor responsiveness to short-term dopaminergic therapy (hereafter, PD non-responders), according to the comparison of their performance in MDS-UPDRS III total in OFF vs. ON condition. The cut-off values for differentiation between subgroups were defined as follows:
(i) PD responders: the minimum percentage change of MDS-UPDRS III from OFF to ON condition $\geq$ 20 % and minimal clinically important improvement on MDS-UPDRS III from OFF to ON condition $\geq$ 4;
(ii) PD non-responders: the maximum percentage change of MDS-UPDRS III from OFF to ON condition < 20 % and maximal clinically important improvement on MDS-UPDRS III from OFF to ON condition $\leq$ 3.

Reporting for specific materials, systems and methods

We require information from authors about some types of materials, experimental systems and methods used in many studies. Here, indicate whether each material, system or method listed is relevant to your study. If you are not sure if a list item applies to your research, read the appropriate section before selecting a response.

Materials & experimental systems

n/a	Involved in the study	
☒	☐	Antibodies
☒	☐	Eukaryotic cell lines
☒	☐	Palaeontology and archaeology
☒	☐	Animals and other organisms
☐	☒	Human research participants
☒	☐	Clinical data
☒	☐	Dual use research of concern

Methods

n/a	Involved in the study	
☒	☐	ChIP-seq
☒	☐	Flow cytometry
☒	☐	MRI-based neuroimaging

Human research participants

Policy information about studies involving human research participants

Population characteristics

See above.

Recruitment	The Parkinson's disease patients were recruited at General University Hospital in Prague. The control subjects were recruited from the general community through advertisements. No selection bias was present.
Ethics oversight	The study was approved by the Ethics Committee of the General University Hospital in Prague, Czech Republic and have therefore been performed in accordance with the ethical standards laid down in the 1964 Declaration of Helsinki and its later amendments. All participants provided written, informed consent to the neurological examination and recording procedure.

Note that full information on the approval of the study protocol must also be provided in the manuscript.
